# Transarterial embolization to treat a massive hemothorax during mechanical circulatory support via puncturing of the extracorporeal membrane oxygenation circuit

**DOI:** 10.1186/s42155-024-00460-8

**Published:** 2024-05-21

**Authors:** Ryota Tsushima, Takaaki Maruhashi, Yutaro Kurihara, Takehiro Hashikata, Yasushi Asari

**Affiliations:** 1 Department of Emergency and Critical Care Medicine, Kitasato University School of Medicine, 1-15-1KitasatoMinami-Ku, Sagamihara-City, Kanagawa-ken 252-0375 Japan; 2 Department of Cardiovascular Medicine, Kitasato University School of Medicine, 1-15-1KitasatoMinami-Ku, Sagamihara-City, Kanagawa-ken 252-0375 Japan

**Keywords:** Impella, Mechanical circulatory support, Transarterial embolization, Venoarterial extracorporeal membrane oxygenation

## Abstract

**Background:**

Current guidelines recommend the use of mechanical circulatory support (MCS) for patients with cardiogenic shock that is refractory to medical therapy. Bleeding is the most common complication of MCS. Transarterial embolization (TAE) is often performed to treat this complication, because it is a less invasive hemostatic procedure. However, the TAE option needs to be carefully considered during MCS, as the access route may be limited during MCS.

**Case presentation:**

A man in his 70 s was diagnosed with acute myocardial infarction and underwent percutaneous coronary intervention via venoarterial extracorporeal membrane oxygenation (VA-ECMO) and Impella. During treatment in the intensive care unit, he suffered damage to a branch of the internal thoracic artery during a cardiac drainage procedure, which was subsequently treated via emergency TAE.

An ECMO return cannula and an Impella sheath were inserted into the patient’s right and left femoral arteries, respectively. An approach from the left brachial artery was selected, and the left internal thoracic artery was embolized. Subsequently, the patient required re-intervention to treat re-bleeding from another artery. Because it was difficult to target the target artery from the brachial one, owing to interference from the Impella catheter, the ECMO circuit near the return cannula was punctured and a guiding sheath was inserted. The ECMO flow and the patient’s blood pressure decreased following placement of this guiding sheath. We were thus able to maintain the patient’s blood pressure by increasing the infusion fluids and Impella flow, and embolize the target artery using a gelatin sponge to achieve hemostasis.

**Conclusion:**

When TAE is difficult to perform during MCS using an approach from the upper extremities, a lower extremity approach with a sheath inserted into the ECMO circuit may represent a viable alternative.

## Background

Patients who experience severe cardiogenic shock that is refractory to drug therapy may require mechanical circulatory support (MCS), such as venoarterial extracorporeal membrane oxygenation (VA-ECMO), intra-aortic balloon pumping (IABP), or Impella® (Abiomed Inc., Danvers, MA, USA). VA-ECMO, which consists of a centrifugal pump and an artificial lung, can assist both cardiopulmonary functions. To initiate VA-ECMO, large-diameter cannulas are inserted into the femoral artery and vein. Blood is drained from the inferior vena cava using a centrifugal pump and oxygenated using an artificial lung. Oxygenated blood is then returned through the femoral artery to assist circulation via retrograde blood flow. However, VA-ECMO alone is an afterload for the patient’s cardiac output that can cause left ventricular enlargement and elevated left ventricular pressure. This can lead to a subsequent recovery of cardiac function.

Impella is a left ventricular assist device that can be percutaneously inserted through the femoral artery. Its tip is placed in the left ventricular cavity, and an axial flow pump draws blood from the left ventricle before injecting it into the aorta. The introduction of Impella leads to immediate and sustained unloading of the left ventricle while increasing overall systemic cardiac output, which promotes the improvement of cardiac function following myocardial infarction. Recently, the effectiveness of VA-ECMO plus Impella (i.e., ECPELLA), has been reported as a strategy wherein each method compensates for the shortcomings of the other [1, 2]. However, it is well known that the introduction of MCS increases the risk of severe bleeding as a potential complication, which is more pronounced in ECPELLA than in VA-ECMO alone [3]. Thus, transarterial embolization (TAE) may be needed to stop cases of severe bleeding during ECPELLA, but the bilateral femoral arteries are already in use and the choice of approach site is, therefore, limited. Herein, we report a case where TAE of the left intercostal artery, approached through the ECMO circuit, was used to treat a massive hemothorax during ECPELLA.

## Case presentation

**Fig. 1:**
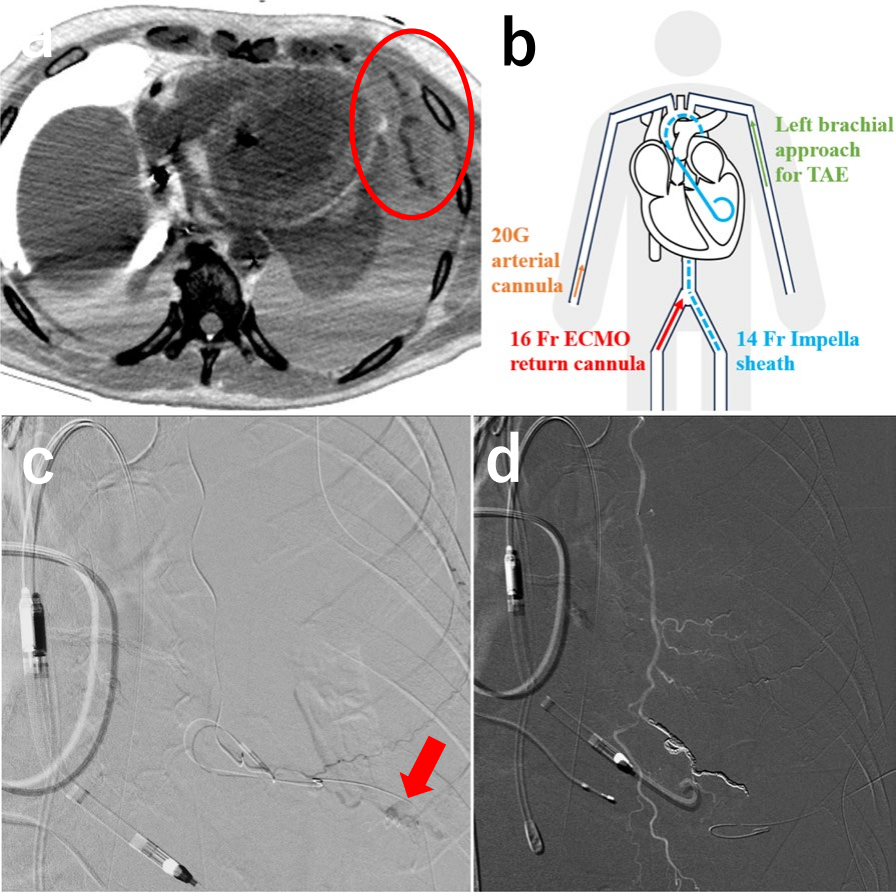
CECT and TAE on the 7th day of the patient’s hospitalization. **a** Arterial phase of thoracic CECT showing a left hemothorax and EV into the left thoracic cavity (circle). **b** The venoarterial extracorporeal membrane oxygenation (VA-ECMO) plus Impella (ECPELLA) circuit and the access route for TAE. **c** Approaching from the left brachial artery, a left internal thoracic angiography showing EV in the peripheral branch (arrow). **d** The bleeding source was embolized using a N-butyl-2-cyanoacrylate-Lipiodol mixture, and its lower intercostal branch was embolized with a metal coil to prevent bleeding from the collateral tract. Final angiography confirmed the disappearance of EV. CECT: chest contrast-enhanced computed tomography, TAE: transarterial embolization, EV: extravasation

**Fig. 2:**
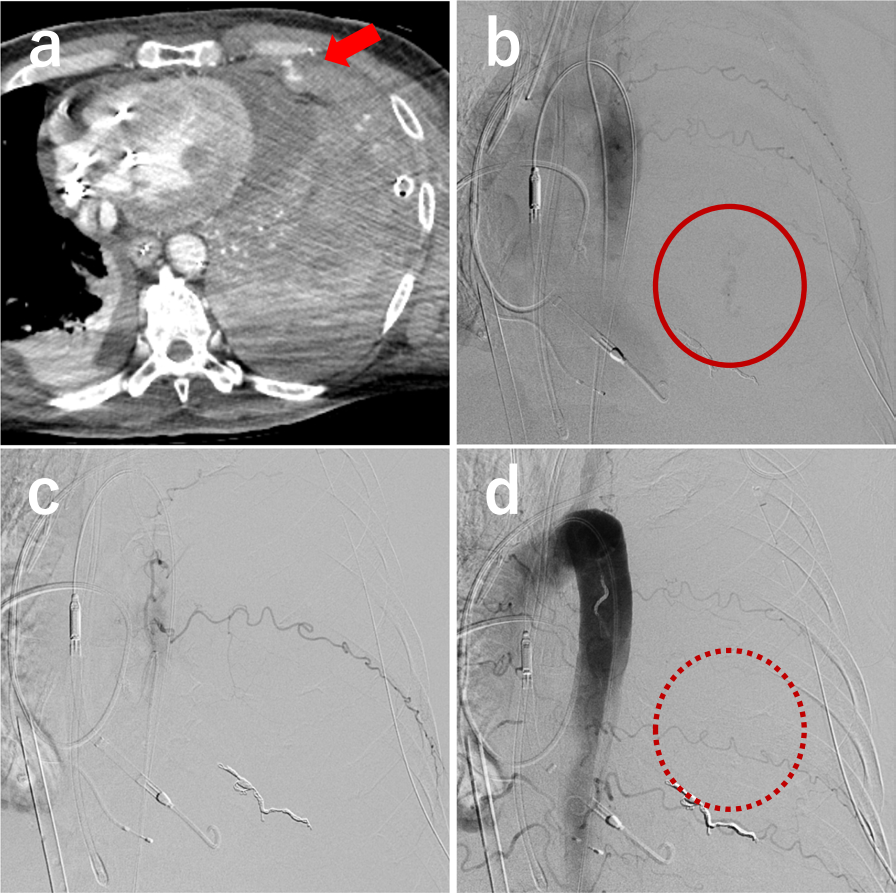
CECT and TAE on the 12th day of the patient’s hospitalization. **a** Arterial phase of a thoracic CECT showing a left hemothorax and residual contrast media EV into the left thoracic cavity (arrow). **b** Aortography with a pigtail catheter advanced from the brachial artery to the descending aorta showing an EV from the 3rd left posterior intercostal artery (circle). **c** Approaching from the ECMO return cannula, the left intercostal artery was targeted and embolized using a gelatin sponge. **d** Final aortography confirming the disappearance of the EV (dotted circle). CECT: chest contrast-enhanced computed tomography, TAE: transarterial embolization, EV: extravasation, ECMO: extracorporeal membrane oxygenation

**Fig. 3:**
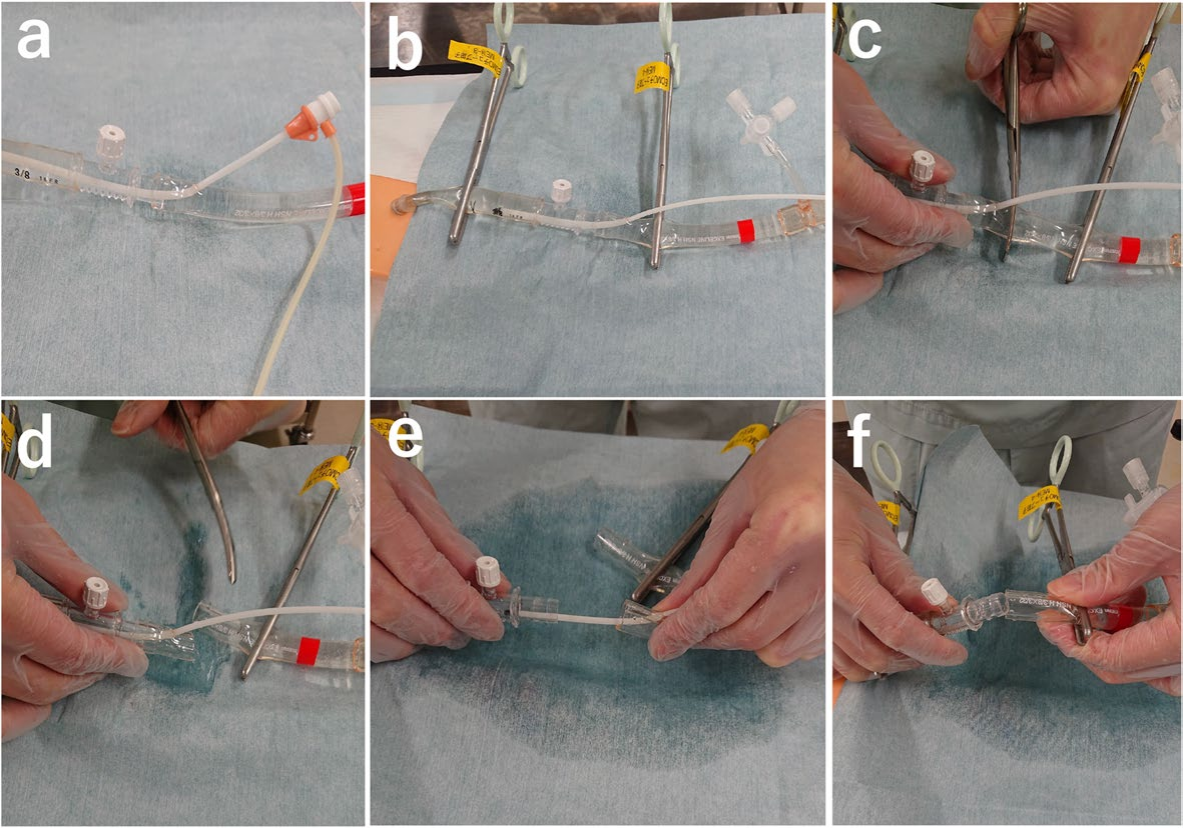
Removal of the sheath after TAE via puncturing of the ECMO circuit (reproduced using a vascular model). **a** The ECMO circuit near the return cannula was punctured and a guiding sheath was inserted using the Seldinger technique. **b** The ECMO flow was temporarily stopped, and the ECMO circuit and return cannula were clamped. **c**,**d** The ECMO circuit near the insertion site of the sheath was cut off. **e** The sheath was removed with the cut-off ECMO circuit. **f **The ECMO circuit and return cannula were reconnected, and ECMO flow was resumed. TAE: transarterial embolization, ECMO: extracorporeal membrane oxygenation

A man in his 70 s was brought to our hospital with chest pain and was diagnosed with acute myocardial infarction. He underwent percutaneous coronary intervention via ECPELLA to treat his hemodynamic instability. Within 1 week following this intervention, pericardial drainage for pericardial effusion and subsequent thoracentesis for pleural effusion were performed via the subcostal approach. This revealed hematogenous pleural effusion, and the ECMO flow became unstable because of drainage insufficiency. Consequently, contrast-enhanced computed tomography (CECT) was performed on the 8th day of the patient’s hospitalization. A large amount of extravasation (EV) of the contrast agent into the left thoracic cavity was observed (Fig. 1a), and we suspected damage to a branch of the adjacent internal thoracic artery caused by the pericardial drainage catheter. These findings indicated a need for emergency TAE. A 16 Fr ECMO return cannula (PCKC-A®; Senko Medical Instrument Mfg. Co., Ltd., Tokyo, Japan) and a 14 Fr Impella sheath were inserted into the right and left femoral arteries, respectively (Fig. 1b). Following this, a 4 Fr introducer sheath (Medikit Super Sheath®; Medikit, Tokyo, Japan) was inserted into the left brachial artery, and a 4 Fr shepherd's hook catheter (NCU; Medikit, Tokyo, Japan) was used to target the left internal thoracic artery. Left internal thoracic angiography revealed an EV in a peripheral branch (Fig. 1c). The microcatheter was advanced to the target artery and embolized using a N-butyl-2-cyanoacrylate (NBCA)-Lipiodol mixture (a NBCA:Lipiodol ratio of 1:3). To prevent bleeding from the collateral artery, its lower intercostal branch was embolized using six metal coils (C-stopper coil® 0.014-inch 100 mm, 2.8 mm; PIOLAX medical device, Kanagawa, Japan; Fig. 1d). However, the hematogenous pleural effusion did not decrease and the patient required frequent blood transfusions. On the 12th day of hospitalization, a repeat CECT revealed residual EV of the contrast agent in the left thoracic cavity (Fig. 2a). An approach from the left brachial artery was selected and the left internal thoracic artery was contrasted as before; however, no EV was observed. A pigtail catheter was advanced from the brachial artery to the descending aorta, and aortography revealed EV from the 3rd left posterior intercostal artery (Fig. 2b). We attempted to select the target artery using various catheters, such as cobra and shepherd’s hook types, but were unable to access the target artery. This may have been due to the bifurcation angle or interference from the Impella catheter. We then decided to approach from the lower extremities, by puncturing the ECMO circuit near the return cannula. A guiding sheath (6 Fr Parent Plus®, 55 cm; MEDIKIT, Tokyo, Japan) and a 5 Fr catheter (SHK-KUSANO®, 80 cm; Hanaco Medicak, Saitama, Japan) were inserted into the ECMO circuit to target the left intercostal artery (Figs. 2c, 3a). After the guiding sheath was placed, the ECMO flow decreased from 3 L/min to 2 L/min, and the patient’s blood pressure decreased from 116/104 mmHg to 76/60 mmHg. We maintained circulatory dynamics by increasing the volume of fluid infusion and the Impella’s performance level. The coaxial technique, using a 1.7 Fr microcatheter (Progreat λ®, 130 cm; Terumo Corp., Tokyo, Japan) and a 0.016-inch guidewire (SUCCEDO®, 180 cm; Boston Scientific, Boston, MA, USA), was used to select the target artery and embolize it using a gelatin sponge. The upper and lower intercostal arteries were embolized in the same manner, and post-TAE aortography confirmed the absence of EV (Fig. 2d). Following TAE, the ECMO flow was temporarily stopped and both the ECMO circuit and return cannula were clamped (Fig. 3b). The ECMO circuit near the insertion site of the sheath was then cut off (Fig. 3c,d), and the sheath was removed using a cut-off ECMO circuit (Fig. 3e). Lastly, the ECMO circuit and return cannula were reconnected (Fig. 3f), and the ECMO flow was resumed. The patient’s anemia did not worsen thereafter, his hematogenous pleural effusion decreased, and hemostasis was achieved (Table 1). Unfortunately, the patient died from multiple organ failure caused by his primary disease on the 33rd day of his hospitalization.

**Table 1:** Changes in hemoglobin levels, transfusion volume, and thoracic drain drainage volume before and after TAE

Clinical data	TAE–1 day	TAE	TAE + 1 day	TAE + 2 days
Hemoglobin levels (g/dL)	6.9	8.4	9.9	9.8
Drainage volume from thoracic drain (mL)	2,420	1,730	820	300
Transfusion volume (mL)				
Red blood cell count	1680	840	560	560
Fresh frozen plasma	1,920	1,200	0	0

*TAE* Transarterial embolization

## Discussion

We report a case wherein TAE was used to treat a massive hemothorax during MCS via puncturing of the ECMO circuit, owing to the difficulty inherent to the use of the typical brachial artery approach during ECMO.

Current guidelines recommend the use of MCS to treat cardiogenic shock that is refractory to medical therapy [4, 5]. Anticoagulation measures are necessary during MCS management, as the most common complication is bleeding. Major bleeding occurs in 31% of patients who undergo ECMO, [6] 31% of patients on Impella, and 16% of patients receiving IABP [7]. Furthermore, bleeding complications are significantly more common during ECPELLA compared to either ECMO or Impella alone [8, 9]. TAE is an effective hemostatic strategy for treating MCS-associated bleeding, owing to the high invasiveness of open hemostatic surgery [10]. However, the limited access routes available during ECPELLA present significant challenges, often making the approach quite difficult. If sheaths or cannulas have been inserted into the bilateral femoral arteries for ECPELLA, the brachial artery approach is the preferred choice; however, subclavian artery occlusion or a dialysis shunt may complicate this approach. In such cases, the ECMO return cannula approach may represent a viable option. In one case report, [11] an emergency TAE was performed for a VA-ECMO cannulation-induced injury of the common iliac artery by reinserting the ECMO return cannula into the contralateral femoral artery and approaching the target artery via the ECMO return cannula. This ECMO-based approach represents a viable option for TAE during MCS management.

The disadvantages of ECMO circuit puncture are as follows: 1) theoretically, ECMO flow decreases after placement of the introducer sheath, owing to increased resistance in the ECMO circuit [11, 12]; 2) the distance to the target artery becomes longer than that in conventional approaches, thus requiring a longer catheter [11]; 3) ECMO flow must be temporarily stopped when the introducer sheath is removed. In a single-center study including 20 patients, wherein the introducer sheath was placed in the ECMO return cannula and coronary angiography was performed; ECMO flow was reduced by more than 15% on average [12]. The same concept applied in our case; however, TAE was safely accomplished by increasing the Impella’s performance level. The patient’s blood pressure did not decrease when the sheath was removed from the return cannula following the TAE.

## Conclusions

Thus, the ECMO approach of puncturing the return cannula represents a viable option for TAE during ECPELLA. When TAEs prove difficult to perform due to the unsuitability of upper extremities access during MCS (e.g., in cases of ECPELLA), a lower extremity approach with a sheath inserted into the ECMO circuit may offer a viable alternative. Notably, ECMO flow is reduced during this procedure, owing to increased resistance in the ECMO circuit, and the distance to the target artery is longer than it is when conventional approaches are used.

## Data Availability

The datasets used and/or analyzed during the current study are available from the corresponding author on reasonable request.

## Abbreviations

MCS: Mechanical circulatory support
TAE: Transarterial embolization
VA-ECMO: Venoarterial extracorporeal membrane oxygenation
IABP: Intra-aortic balloon pumping
ECPELLA: VA-ECMO plus Impella
CECT: Contrast-enhanced computed tomography
EV: Extravasation
NBCA: N-butyl-2-cyanoacrylate
